# CT Morphometric Analysis to Determine the Anatomical Basis for the Use of Transpedicular Screws during Reconstruction and Fixations of Anterior Cervical Vertebrae

**DOI:** 10.1371/journal.pone.0081159

**Published:** 2013-12-11

**Authors:** Chun Chen, Dike Ruan, Changfu Wu, Weidong Wu, Peidong Sun, Yuanzhi Zhang, Jigong Wu, Sheng Lu, Jun Ouyang

**Affiliations:** 1 Department of Anatomy, Southern Medical University and Guangdong Provincial Medical Biomechanical Key Laboratory and Academy of Orthopedics of Guangdong Province, Guangzhou, Guangdong, China; 2 Department of Orthopedic Surgery, The Navy General Hospital, Southern Medical University, Beijing, China; 3 The Third School of Clinical Medicine, Southern Medical University, Guangzhou, Guangdong, China; 4 Department of Orthopedic Surgery, Affiliated Hospital of Inner Mongolia Medical College, Inner Mongolia, China; 5 Department of Orthopedic Surgery, The 306th Hospital of People's Liberation Army, Beijing, China; 6 Department of Orthopedic Surgery, Kunming General Hospital, Kunming, Yunnan, China; 7 Shenzhen Digital Orthopedic Engineering Laboratory, Shenzhen, Guangdong, China; University Medical Center (UMC) Utrecht, The Netherlands

## Abstract

**Background:**

Accurate placement of pedicle screw during Anterior Transpedicular Screw fixation (ATPS) in cervical spine depends on accurate anatomical knowledge of the vertebrae. However, little is known of the morphometric characteristics of cervical vertebrae in Chinese population.

**Methods:**

Three-dimensional reconstructions of CT images were performed for 80 cases. The anatomic data and screw fixation parameters for ATPS fixation were measured using the Mimics software.

**Findings:**

The overall mean OPW, OPH and PAL ranged from 5.81 to 7.49 mm, 7.77 to 8.69 mm, and 33.40 to 31.13 mm separately, and SPA was 93.54 to 109.36 degrees from C3 to C6, 104.99 degrees at C7, whereas, 49.00 to 32.26 degrees from C4 to C7, 46.79 degrees at C3 (TPA). Dl/rSIP had an increasing trend away from upper endplate with mean value from 1.87 to 5.83 mm. Dl/rTIP was located at the lateral portion of the anterior cortex of vertebrae for C3 to C5 and ipsilateral for C6 to C7 with mean value from −2.70 to −3.00 mm, and 0.17 to 3.18 mm. The entrance points for pedicular screw insertion for C3 to C5 and C6 to C7 were recommended −2∼−3 mm and 0–4 mm from the median sagittal plane, respectively, 1–4 mm and 5–6 mm from the upper endplate, with TPA being 46.79–49.00 degrees and 40.89–32.26 degrees, respectively, and SPA being 93.54–106.69 degrees and 109.36–104.99 degrees, respectively. The pedicle screw insertion diameter was recommended 3.5 mm (C3 and C4), 4.0 mm (C5 to C7), and the pedicle axial length was 21–24 mm for C3 to C7 for both genders. However, the ATPS insertion in C3 should be individualized given its relatively small anatomical dimensions.

**Conclusions:**

The data provided a morphometric basis for the ATPS fixation technique in lower cervical fixation. It will help in preoperative planning and execution of this surgery.

## Introduction

Cervical spine injury, instability, degenerative diseases, cancer, osteoporosis and other pathological diseases affecting anterior vertebral bodies are commonly encountered by spinal surgeons [1]. Anterior cervical inter-body fusion (ACIF), posterior cervical pedicle screw (pCPS) and lateral mass screw (LMS) are the most commonly performed surgical interventions and have consistently acceptable results [2]–[7]. Despite acceptable results with the use of both techniques, the number of complications and failures when compared to surgeries in other region are relatively higher [8]–[10]. In many cases both anterior and posterior approaches are employed and can be named circumferential surgery as in global fusion for tumor radical excisions [4]. Improved implant design, metallurgy and biomechanical analysis of failures, have led to development of newer fixation devices and surgical techniques especially for cases involving multiple segment fixation and patients with osteoporosis [11]. Circumferential revision involving global fusion surgery can effectively solve the problem and improve stability, but a second surgery increases the patient's morbidity and adds to the cost of treatment significantly.

It is necessary that we devise unique methods of fixation that have all the advantages of circumferential surgery without of the disadvantages of increased morbidity and cost. Koller et al [12] described anterior cervical transpedicular screw (ATPS) fixation to solve the above problems in 2008 and reported his results from a study on morphological feasibility, indications, and technical prerequisites for the same. Many studies have shown that there are a great degree of morphological differences between the Asian and European/American populations [13], especially in the femur [14]–[16] and cervical vertebrae regions [13], [17], [18]. In order to achieve optimal surgical outcomes, it is therefore imperative that pertinent anatomical data, especially with regard to pedicles and vertebral bodies, be obtained. This study was done to measure morphometric parameters of different cervical vertebrae in the Chinese population using Mimics image processing software.

## Materials and Methods

### Ethics statement

The study protocol was ethically approved by the Human Research Ethics Committee of Kunming Military General Hospital (Yunnan) and Human Research Ethics Committee of the 306th Hospital of People's Liberation Army (Beijing) and Human Research Ethics Committee of Affiliated Hospital of Inner Mongolia Medical College (Inner Mongolia). Prior written informed consent was obtained from all study participants.

### Sample collection and measurement method

**Figure 1:**
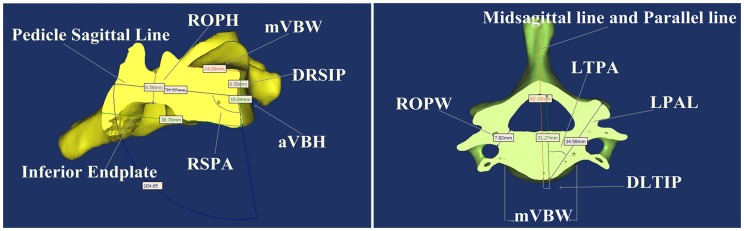
Morphological parameters in Mimics software relating to Table 1.

The study consisted of 80 patients (35 females and 45 males), who underwent cervical CT examination in Kunming Military General Hospital, the 306th Hospital of People's Liberation Army and Affiliated Hospital of Inner Mongolia Medical College during a period lasting from June 1, 2011, to July 30, 2012. The mean age for all patients was 52.51 years (range 25–76 years), the mean age of patients was 49.14 years for males and 53.3 years for females. None of the patients had any evidence of infectious, neoplastic, traumatic, or degenerative diseases involving the spine, or any evidence of congenital or developmental spinal malformation. All patients were scanned using a helical CT scanner (Somatom Sensation 64, Siemens Medical Solutions, Erlangen, Germany). Because proper slice thickness is important for the performance of high quality multi-planar reformation, a skilled technician and appropriate imaging apparatus were used. The primary DICOM images were acquired using a standard algorithm with 1.25 mm slice thicknesses and 0.6 mm reconstruction intervals. Reconstructions were performed using Mimics 14.11 (Materialise Corp., Leuven, Belgium). Before measurements were made and the data collected, the calibrated phantom model had to be established. As shown in Fig. 1,2 3, the DICOM images from the regions of interest are converted to 3-D surface models using an adapted marching cubes algorithm that takes the partial volume effect into account, leading to highly accurate 3-D models on which measurements can be performed. An interactive image processing strategy (“Threshold” and “Region growth”) was used to segment the contours of each vertebra. In this study, a lower threshold of 226 Hounsfield units and an upper threshold of 3071 Hounsfield units were used. The 3-D reconstruction of each vertebra can be freely translated and rotated. The vertebral anatomic structure (such as, left and right anterior or posterior part of unicinate process, left and right medial edge of transverse foramen, and anterior or posterior mid-sagittal line of vertebral) had to be defined. For pedicle measurement, a degree of cutting accuracy will depend on fitting the center line of the pedicle of a 3-D model which was determined by pedicle curvature. The vertebral pedicle length, width and depth were measured on the cutting plane, and sagittal or coronal sections views were rotated to ensure that the computation of axial line of pedicle was located at the central cervical pedicle by visual observation. After vertebrae cutting, the specific parameters [12] used during the measuring process are illustrated in Table 1 and Fig. 1. The detailed following parameters were assessed: aVBH (anterior Vertebral Body Height), mVBD (midbody Vertebral Body Depth), mVBW (midbody Vertebral Body Width), l/rOPW (left/right Outer Pedicle Width), l/rOPH (left/right Outer Pedicle Height),l/rTPA (left/right Transverse Pedicle Angle), l/rSPA (left/right Sagittal Pedicle Angle), l/rPAL (left/right Pedicle Axis Length), l/rTIP (left/right Transverse Intersection Point), Dl/rTIP (Distance left/right Transverse Intersection Point), l/rSIP (left/right Sagittal Intersection Point), Dl/rSIP (Distance left/right Sagittal Intersection Point).

**Figure 2:**
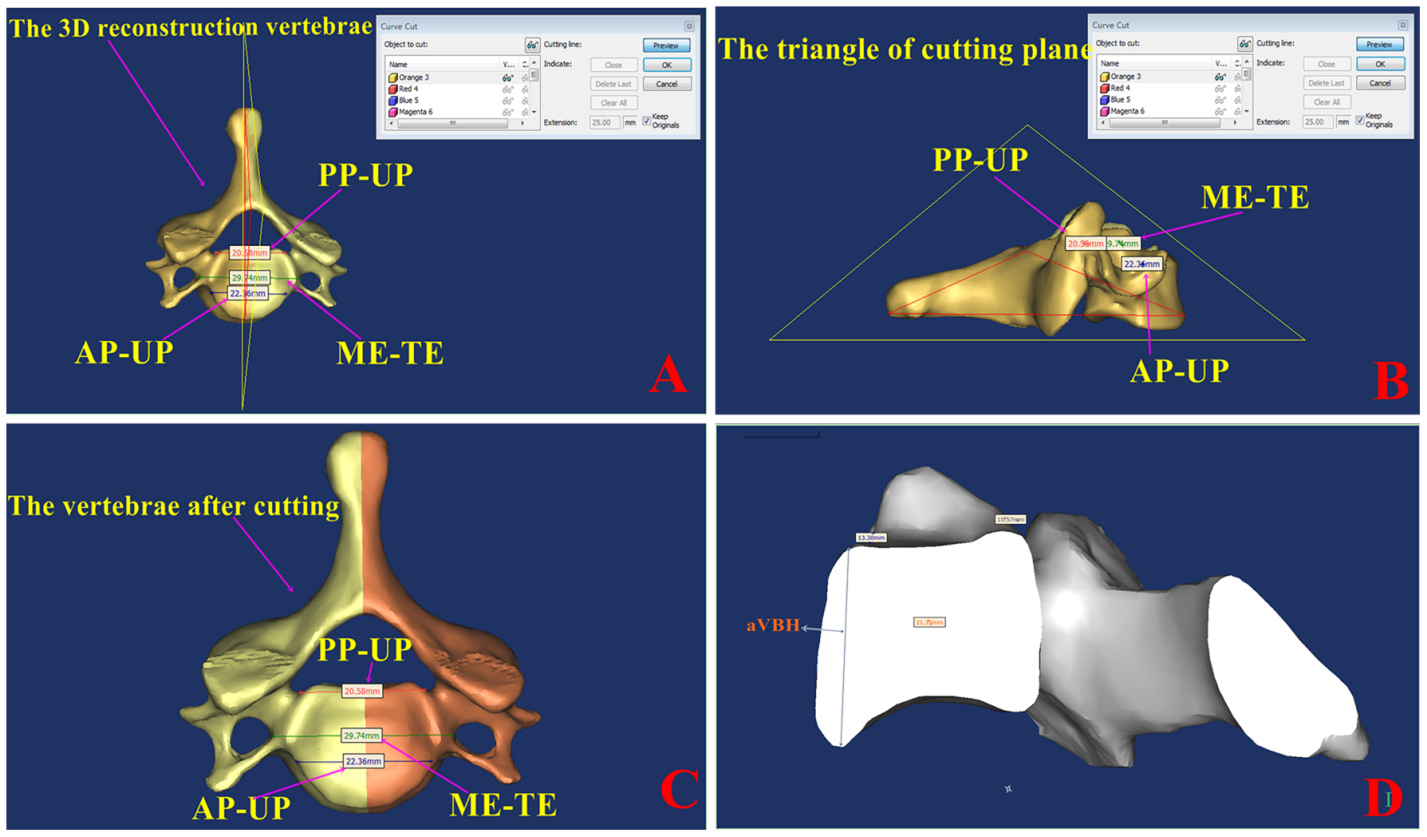
The process of part of measuring parameters using Mimics 14.11 software. A: The target vertebra was chosen in Mimics 14.11 software. Three lines (AP-UP, ME-TE, PP-UP) were drawn and the cutting plane was adopted with the midpoint of the three lines. 1, AP-UP: The line between left and right anterior part of unicinate process. 2, ME-TF: The line between left and right medial edge of transverse foramen. 3, PP-UP: The line between left and right posterior part of unicinate process. B: Profile of the cutting plane can be observed by rotation. C: The cutting vertebral body was colored. D: The aVBH was measured.

**Figure 3:**
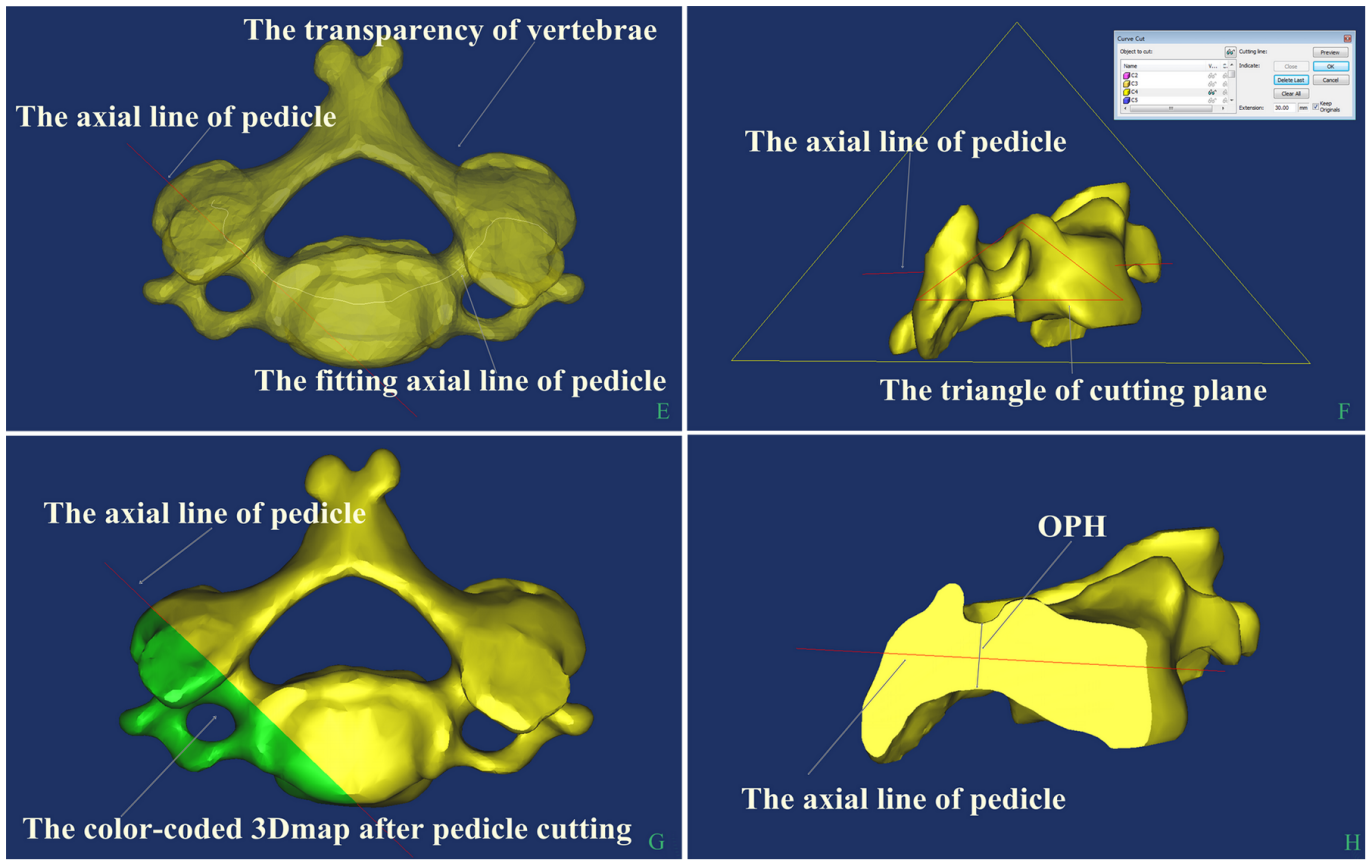
The process of OPH measurement. E: The red fitting axial line of pedicle was computed by pedicle curvature using Mimics software and can be observed by transparency. F: Profile of the cutting plane and can be observed by rotation. G: The cutting pedicle was colored. H: The OPH was measured.

**Table 1:** Cervical anterior transpedicular screw fixation anatomical parameters.

Parameter	Measurement	Description
aVBH	Anterior Vertebral Body Height	Distance cephalad to caudad endplate at mid-sagittal line
mVBD	Midbody Vertebral Body Depth	Antero-posterior vertebral body depth at mid-sagittal line
mVBW	Midbody Vertebral Body Width	Transverse distance from left to right border of vertebral body at mid-vertebral line
l/rOPW	Left/right Outer Pedicle Width	Distance from medial border of transverse foramen to medial border of pedicle
l/rOPH	Left/Right Outer Pedicle Height	Distance from upper to lower pedicle surface in sagittal plane
l/rTPA	Left/Right Transverse Pedicle Angle	Angle formed between transverse pedicle axis and mid-sagittal line
l/rSPA	Left/Right Sagittal Pedicle Angle	Angle formed between plane of anterior vertebral body wall at mid-sagittal line and sagittal pedicle axis
l/rPAL	Left/Right Pedicle Axis length	Distance from anterior vertebral body wall to posterior margin of lateral mass along the transverse pedicle axis
l/rTIP	Left/Right transverse Intersection Point	Transverse intersection point of transverse pedicle axis with anterior vertebral body wall
Dl/rTIP	Distance left/right transverse Intersection Point	Distance between transverse intersection point and mid-sagittal line at the anterior vertebral body wall at each cervical level C3–C7
l/rSIP	Left/Right sagittal Intersection Point	Sagittal intersection point of sagittal pedicle axis with anterior vertebral body wall
Dl/rSIP	Distance left/right sagittal Intersection Point	Distance between sagittal intersection points and cephalad endplate at each cervical level C3 to C7.

[12]. Reproduced with permission from Koller

One researcher measured all the data by Mimics workstation. Linear and angular measurements were done from C3 to C7. All parameters were measured three times by the first author (C.C), and the mean was used as the final value [2], [17].

Considering a three-dimensional screw entrance point, it can be observed that in sagittal plane, the lSIP and rSIP, and in transverse plane, lTIP and rTIP, respectively, resemble conceivable entry points for ATPS into the left and right pedicles. The entry points resemble the projection of the center of a corridor formed by the cervical pedicles onto the anterior vertebral cortex, both in the coronal and sagittal plane. Concurrently with the l/rSIP, mean data of l/rTIP at the levels C3 to C7 were visualized to assess their importance during insertion of ATPS for all levels except C2 [12], [19]. The distances between sagittal intersection points were measured along the anterior cervical column and could be angled and interrupted at the superior and inferior corner of each vertebral body using a polygon measuring tool. Regarding measurements of l/rTIP, those pedicle axes which crossed the mid-sagittal line were scaled as ‘positive’ values, and those intersecting the anterior vertebral body lateral to the mid-sagittal line were scaled as ‘negative’ values. The method of measurement was adapted from the technique reported by Koller [3], [12], [19], [20].

### Data collection and statistical analysis

The ranges, means and standard deviations (Mean±SD) for each parameter were calculated. To compare between the right and left pedicles, all paired structures of the vertebrae were measured individually and an independent samples t-test was performed with the significance set at 95% confidence level. Post-hoc comparisons were done to compare factor levels. Statistical analyses were performed using SPSS 19.0 software (SPSS, Chicago, Illinois, USA).

## Results

The CT data pool comprised 400 cervical vertebrae. All parameters were measured and described in Tables 2, 3, 4. Thirteen linear and four angular parameters wcere measured in cervical vertebrae. The resultant P<0.05 showed that all linear and angular measurements were significant statistically in any parameter.

**Table 2:** Linear parameters measured of the aVBH, mVBD and mVBW (Mean± SD, mm).

Cervical level	aVBH			mVBD			mVBW		
	Male Range	Female Range	All Range	Male Range	Female Range	All Range	Male Range	Female Range	All Range
C3	14.16±1.61	13.45±0.99	13.94±1.47	17.41±1.90	15.04±1.20	16.67±2.03	20.63±1.83	19.57±1.93	20.30±1.91
	9.41–16.27	11.67–15.27	9.41–16.27	12.65–21.43	12.65–16.71	12.65–21.43	15.67–24.63	16.76–23.32	15.67–24.63
C4	14.00±1.62	12.90±0.71	13.67±1.49	17.47±1.90	14.85±1.35	16.69±2.12	21.45±2.03	20.25±1.70	21.09±2.00
	8.44–16.21	11.56–14.06	8.44–16.21	12.20–21.93	11.24–16.85	11.24–21.93	16.15–25.38	17.87–23.19	16.15–25.38
C5	13.13±1.59	12.04±1.07	12.80±1.52	17.07±1.69	15.16±2.12	16.50±2.01	22.51±2.31	21.09±2.21	22.08±2.35
	9.53–15.35	10.48–14.57	9.53–15.35	12.48–21.04	11.35–20.55	11.35–21.04	17.04–27.88	16.95–25.19	16.95–27.88
C6	13.22±1.67	12.21±0.96	12.94±1.56	17.36±1.79	15.16±1.99	16.74±2.08	24.37±2.11	23.78±2.11	24.20±2.10
	8.27–16.78	10.46–14.22	8.27–16.78	13.49–21.81	11.69–18.56	11.69–21.81	20.70–29.07	21.28–27.62	20.70–29.07
C7	14.61±1.59	13.73±1.05	14.35±1.49	17.00±1.57	15.17±1.91	16.45±1.86	27.71±2.44	26.30±2.06	27.29±2.40
	10.22–17.01	12.37–15.36	10.22–17.01	13.68–20.69	11.37–17.95	11.37–20.69	21.19–32.78	23.66–29.18	21.19–32.78

**Table 3:** Linear parameters for pedicle characterization (Mean±SD, mm).

Cervical level		OPW		OPH		PAL	
		left Range	right Range	left Range	right Range	left Range	right Range
C3	Male	6.02±0.74	6.14±0.84	8.09±0.94	7.92±0.99	34.39±1.70	34.19±1.95
		4.68–7.60	4.59–8.42	5.81–9.71	5.95–10.96	30.25–37.56	28.45–38.01
	Female	5.34±0.60	5.09±0.77	7.26±0.70	7.29±0.74	31.26±1.70	31.61±1.14
		4.55–6.46	4.38–7.09	6.41–8.55	5.85–8.36	29.09–34.33	30.04–33.41
	All	5.81±0.85		7.77±0.94		33.40±2.15	
C4	Male	6.08±0.69	6.19±0.82	8.85±1.12	8.73±1.01	34.83±2.24	34.65±2.08
		4.41–7.70	4.50–8.05	6.60–11.20	5.80–10.85	29.20–38.90	29.61–38.35
	Female	5.35±0.58	5.16±0.60	7.87±0.73&	7.51±0.71	31.97±2.28	32.35±2.47
		4.22–6.10	4.32–6.19	6.66–8.90	6.26–8.70	28.28–35.74	28.43–35.86
	All	5.87±0.81		8.46±1.09		33.97±2.49	
C5	Male	6.43±0.88	6.60±0.83	8.55±1.25	8.57±1.20	35.43±3.19	35.87±2.90
		4.83–8.29	4.62–8.29	5.90–10.84	5.67–11.20	27.00–42.64	30.65–43.60
	Female	5.86±0.80	5.86±0.73	7.49±0.69	7.27±0.46	32.46±2.12	33.56±3.04
		4.83–7.87	4.54–7.33	6.26–8.92	6.57–8.39	29.06–35.86	28.44–39.73
	All	6.31±0.87		8.20±1.19		34.84±3.14	
C6	Male	6.91±0.761	6.99±0.71	8.45±1.11	8.35±1.32	34.40±3.47	34.63±2.96
		5.44–8.77	5.86–8.67	6.65–11.06	5.98–11.09	25.64–40.62	28.96–40.54
	Female	6.18±0.86	6.19±0.86	7.58±0.89	7.68±0.71	31.77±2.78	32.62±2.67#
		4.93–8.21	4.97–7.65	6.64–9.06	7.01–8.98	28.44–35.32	29.03–36.33
	All	6.73±0.83		8.18±1.15		33.85±3.21	
C7	Male	7.55±0.90	7.67±0.89	8.93±1.15∧	8.97±1.32	30.56±2.50∧	32.36±2.16
		5.91–8.94	5.58–9.86	6.27–11.34	5.85–12.44	25.62–34.09	27.71–35.66
	Female	6.19±0.86Δ	7.14±0.50Δ	8.12±0.97	8.04±0.74	29.49±2.48Δ &	31.02±1.98Δ &
		6.04–8.60	6.40–8.09	6.68–9.32	7.01–9.05	25.37–32.54	27.59–34.22
	All	7.49±0.85		8.69±1.19		31.13±2.50	

^#^ Compared with rPAL between males and females in C6; P>0.05,

Compared with l/rOPW and l/rPAL between males and females in C7, P>0.05,

Statistically significant differences in males of l/rPAL (C7); P<0.05,

^&^ Statistically significant differences in females of l/rPAL (C7) P<0.05.

**Table 4:** Linear parameters and angular measurements for vertebral characterization (Mean±SD).

Cerv ical level		TPA(degrees)		SPA(degrees)		DTIP(mm)		DSIP(mm)	
		left Range	right Range	left Range	right Range	left Range	right Range	left Range	right Range
C3	Male	48.56±4.35∧	46.16±3.65∧	93.33±7.84	92.29±7.29	−3.71±2.28	−3.04±1.93	2.18±1.28	1.76±0.80
		38.46–60.99	41.06–55.64	7 3.65–112.29	76.29–106.0	−8.83–2.33	−8.56–2.55	0.20–5.67	0.10–3.28
	Female	46.78±3.40*	44.28±3.88*	93.39±3.16*	93.92±3.64*	−1.83±0.98*	−1.56±1.77*	1.70±0.69*	1.64±0.71*
		4.26–51.95	39.33–53.80	86.17–113.31	87.73–99.12	−3.32–0.00	−4.92–1.10	0.71–2.77	0.10–2.65
	All	46.79±4.11		93.54.97±6.91		−2.70±2.50		1.87±0.98	
C4	Male	51.08±5.17∧	47.79±5.33∧	99.22±9 .18	98.58±9.49	−4.45±2.57	−3.83±2.80	2.65±1.36	2.90±1.42
		38.93–69.14	31.39–56.05	83.06–126.85	76.71–116.47	−10.84–2.42	−7.77–3.96	0.22–6.43	0.00–6.71
	Female	49.25±5.6	46.72±5.58	100.36±7.30	97.85±7.26	−3.00±2.69	−2.53±2.85	2.80±1.11	2.39±0.89
		39.98–61.47	38.63–57.11	88.59–110.03	84.14–105.26	−6.47–2.73	−8.66–2.18	0.96–4.46	0.57–3.83
	All	49.00±5.53		98.95±8.68		−3.73±2.75		2.72±1.28	
C5	Male	48.58±6.43	47.56±7.72	105.51±7.74	108.59±8.40	−3.70±3.54	−3.22±2.64	4.00 ±1.41∧	4.48±1.66∧
		34.16–60.04	35.38–80.19	86.41–117.04	89.73–130.32	−10.85–5.55	−6.83–3.01	1.48–7.72	2.08–8.69
	Female	47.04±4.65	45.70±5.08	105.16±11.25	106.61±10.14	−2.07±2.57	−2.25±2.96	3.37±1.34	3.77±1.88
		38.32–53.48	34.03–52.85	86.09–129.69	85.52–124.37	−6.47–2.99	−7.07–3.82	1.56–5.64	1.47–8.51
	All	47.55±6.48		106.69±8.90		−3.00±3.03		4.03±1.59	
C6	Male	41.37±8.32	40.54±6.35	108.63±8.69	109.15±7.53	0.13±4.49	0.02±3.49	5.79±2.46	5.64±2.03
		29.86–58.75	25.48–56.57	96.86–133.63	96.21–126.24	−6.10–7.68	−4.91–8.56	2.35–11.82	2.67–10.88
	Female	40.27±5.81	41.25±5.61	110.45±6.57	110.66±8.57	1.10±2.46	−0.22±2.91	5.35±1.28	5.46±1.27
		32.08–49.59	30.12–51.91	100.28–118.64	99.19–129.63	−2.93–3.90	−6.61–4.50	3.81–8.13	3.79–8.04
	All	40.89±6.86		109.36±7.88		0.17±3.65		5.62±1.99	
C7	Male	32.71±4.41	31.92±2.69	104.98±8.32	104.02±7.38	3.59±2.12	2.77±1.75	5.78±1.14	5.82±1.45
		25.96–41.22	26.80–36.15	92.99–130.57	91.61–118.25	0.00–8.81	0.00–6.59	3.98–7.93	2.88–8.88
	Female	30.88±4.26&	33.39±3.16&	108.20±6.74	104.11±4.05	4.01±1.95	2.41±1.25	5.82±1.45&	5.98±1.26
		23.99–39.14	26.44–38.26	93.74–115.00	99.07–112.89	1.20–7.53	0.92–5.58	3.49–8.90	4.60–8.68
	All	32.26±3.68		104.99±7.26		3.18±1.90		5.83±1.29	

Compared with l/rTPA, l/rSPA, l/rDSIP, l/rDTIP between males and females in C3, P<0.05;

Statistically significant differences in males of l/rTPA (C3, C4), and l/rDSIP (C5), P<0.05,

^&^ Statistically significant differences in females of l/rTPA(C7) and l/rDSIP (C7) P<0.05.

### Linear measurements

There were statistically significant interlevel differences in mVBD between male and female patients (P<0.05). However, statistically differences were found between C5 and C6, C6 and C7 for both gender in mVBW, aVBH (P<0.05). There was a tendency to increase from C3 to C7 for mVBW (Table 2).

There were significant differences between left and right sides of C7 in males and females concerning PAL (P<0.05). Measurements of PAL showed interlevel statistically significant differences between the level C7 and C3 to C6 (P<0.05) irrespective of genders or sides. No statistically significant differences were found for PAL between males and females. There was a similar length of PAL from C3 to C6 except for C7. The lowest value in our measurement was no less than 25 mm in males or females (Table 3).

No significant left or right differences in OPW were found for any of the patients (Table 3). However, merging left and right OPW data, gender as well as vertebral level showed to be a statistically significant factor (P<0.000, P<0.000). The OPW showed a tendency to increase from C3 to C7 (5.81–7.49 mm). Statistically significant differences (P<0.05) were found between males and females in all levels except for C7. No significant differences in males were observed between OPW of C3 and C4, C4 and C5 except for other interlevel comparison (P<0.05). In females, statistically significant differences (P<0.05) were identified in all of the interlevel except for between C3 and C4. Taken together, the mean left and right OPW of the entire group, no statistically significant differences were only found between C3 and C4. The frequency of OPW below 5 mm was 18.89% at C3, 13.82% at C4, 4.34% at C5, 2.56% at C6 and 0% at C7, as well as the frequency of OPW below 4 mm was 0% (Table 3).

Concerning OPH of all patients, there were no significant differences between left and right sides, however gender and vertebral level proved to be a statistically significant factor (P<0.000, P<0.000). Statistically significant differences (P<0.05) were found between males and females in all levels. Statistical analysis revealed significant differences (P<0.05) in males between OPH of C3 and C5 to C6, C6 and C7. In females, significant differences (P<0.05) were observed between C3 and C7, C5 and C7. Merging males and females, a statistically significant interlevel difference (p<0.05) existed between C3 and C4 to C7 (Table 3). The diameters of all ranges were no less than 5.5 mm in all patients (Table 3).

### Angular measurements

With the TPA and SPA (C3–C7), there were no significant gender- or side-related differences except at C3 level (P<0.05). Merging males and females, there was a tendency for an increase of TPA from C3 to C4 (46.79–49.00 degrees) with a reversal of that increase from C5 to C7 (47.55–32.26 degrees), as well as SPA from cephalad C3 to caudad C6 (93.54–109.36 degrees) which subsequently slightly decreased from C6 to C7 (109.36–104.99 degrees). No statistically significant interlevel differences of TPA calculated from merged data of all 80 patients. Significant differences (P<0.05) were observed between TPA of C6 or C7 and C3 to C5, then, SPA of C3 and C6 to C7 for both genders, respectively (Table 4).

### Intersection points

DTIP and DSIP determine the space in the anterior vertebral body for pedicle screws. In measuring distances between lTIP or rTIP and the mid-sagittal line (Dl/rTIP) at the maximum was at C7 in males (8.81 mm) and the minimum was at C5 in males (−10.85 mm, Table 4). Mean distances from adjacent cephalad endplates and mid-sagittal line to the sagittal and transverse intersection points were compiled in Table 4. Dl/rSIP had an increasing trend away from upper endplate (1.87–5.83 mm) and Dl/rTIP had a trend of contralateral turning ipsilateral in C3 to C7 (−2.70∼3.18 mm, Tables 4). There were no significant male versus female, or left versus right differences detected. Merging all the data, no statistically differences of Dl/rTIP was found between C3 and C4 to C5, and between C4 and C5 (Table 4). Using anatomical trajectories of pedicle axis for measuring the l/rSIP, the frequency of these l/rSIP with a distance below 3 mm to its adjacent cephalad disc spaces was 92.22% at C3, 63.82% at C4, 27.17% at C5, 6.41% at C6, and 1.35% at C7. Clinically there is a wider corridor in the sagittal plane to place a 3.5 mm screw inside the pedicles and sufficiently beneath adjacent disc spaces but this is significantly diminished at the level of C3. The pedicle axes intersect each other in the anterior part of the vertebral body in C3 to C5. The mean distances measured between the midsagittal line and l/rTIP shifts slightly from the contralateral to the midsagittal line of the pedicle axis in C3 to C5 and towards the ipsilateral side in C6 to C7. Mostly, pedicle axes that did not cross the midsagittal line at the anterior vertebral body wall were observed at the caudal C6 and C7.

## Discussion

### Indications and advantages of ATPS

Transpedicular screw fixation is effective in the stabilization of the middle and lower cervical spine in cases of vertebral trauma, fractures, deformities, and degenerative disorders. Of the numerous techniques for stabilizing the cervical spine, this fixation method confers superior pullout strength and constructs rigidity. Abumi et al. [21] released a preliminary report on performing cervical transpedicular screw fixation for traumatic dislocations and fractures of the middle and lower cervical spinal column. However, quite a few patients needed multi-segment anterior decompression, and some also required long strut grafts or cages for the reconstruction, thus, were biomechanically inferior and vulnerable to failure, and subsequently revised [9]. Koller et al [8] reported a high non-union rate of 20–50% and net failure rates of 30–100% in multi-segment ACIF. The need for posterior supplemental stabilization was 10–15% in most studies that reviewed multi-segment ACIF [22]. Posterior pedicle fixation for primary or metastatic cervical tumors or cervical osteoporosis with multi-segment fixation may enhance the stability and improve fusion rates, but suffers with its own set of disadvantages. For example, surgical morbidity and rates of infection increase. Excessive intraoperative stripping of paraspinal muscles during posterior cervical fixations can cause long-term neck pain.

Recently, several studies have been aimed at delineating the three-dimensional anatomy of the cervical pedicle and investigating the feasibility of transpedicular fixation in the subaxial cervical spine in an attempt to provide three-column fixation via one approach [12], [23]–[25]. Koller et al [12] reported a new concept (ATPS) that combined the advantages of an anterior approach and superior biomechanical characteristics of cervical pedicle screw fixation, with excellent results. There are, however, few clinical reports since Koller's reported [20], [26]. Yukawa et al [20] found that postoperative lordosis improvement and early bony union occurred in all cases after ATPS. There were no serious complications except for two cases of dysphagia at the final follow-up (mean 12.2 months), and no pedicle perforation. The author thought ATPS is useful in selected cases of multi-segmental anterior reconstruction of the cervical spine with adequate familiarity and experience. Xu et al [26] developed ATPS for five patients with cervical fractures and dislocations in 2011. All of the screw placements were optimal and no pedicle screw perforation was observed. There were no leakages of cerebrospinal fluid and incisions were healing at the final follow-up (mean 10.6 months). There was no dysphagia in Xu's study; the author thought the benefit was secondary to avoidance of abrasive drilling part during the creation of an entrance point and also because of deeper screw placement.

In contrast to traditional anterior fixation, the surgery involving ATPS is technically demanding because the margin of error while placing the screw is very narrow given its proximity to vital structures such as the vertebral artery, upper-lower intervertebral disc and vulnerable neural and vascular structures [11], [27], [28]. Therefore, a thorough 3-D understanding of the vertebrae and pedicle morphology and regional anatomy is required for the accurate identification of the ideal pedicle screw entry, trajectory and screw size. There is substantial data on the pedicle anatomy and various morphometric features of cervical vertebrae for the western population [12], [13], [29]. Previous studies from Korea, the Indian subcontinent and studies from different regions (e.g. hip joints of Chinese population) have shown considerable variation from the western world [24], [26], [30]. There is paucity of similar data on the Chinese population that will enable spinal surgeons from the region to accurately plan their surgical procedures [26], [31]. This study helped to obtain necessary data and establish a baseline for future clinical study of the surgical outcomes.

Mimics software helps to create 3-D models from stacks of 2-D data which can accurately reflected the morphometry of the cervical region. Compared with conventional CT scan measurement, the full size, shape and morphological variables of the pedicle can be directly determined using 3-D reconstruction images. Complex and irregular structures of component elements of cervical vertebrae-pedicle-lamina make conventional CT scan measurement difficult. During scanning, inaccuracy of measurements is created by variable discrimination between bone and soft tissues or choosing the wrong middle layer. In contrast, Mimics is based on 3-D slicing segmentation. There is a better separation delimiter and compensation for a certain degree of scanning defects, which are based on the relationship between the data of gray values among slices and spatial location. Selection errors were made due to the lack of a holistic three dimensional view for selecting the narrowest pedicle on traditional CT scan. A degree of cutting accuracy can be achieved by fitting the center line of the pedicle of a 3-D model, measuring the center line of curvature, and calculating the axial line, then cutting plane at the three midpoints of the lines. Visual observation can be rotated for vertebrae and middle cutting with sagittal or coronal sections by measuring vertebral body and pedicle dimensions (Figures 2 and 3).

### The anatomical character of ATPS related measurements

Morphometric measurements based on CT scans are more efficient in determining pedicle dimensions than manual calliper measurements [23], [25], [32]. CT scans may be able to avoid possible deviations in disc height by post-mortem changes such as dehydration and altered tonus of the soft tissue [33]. Previous studies targeted areas at the coronal or sagittal planes for spiral CT [12], [33], [34]. Although it can display the character of the vertebral anatomy, subjective selection error always appears due to deficiency of scan precision and choice of target area. Mimics software is compatible with data of various types of machines (e.g. CT or MRI) and 3-D reconstruction, region segmentation, output conversion, surface meshing, body meshing and processing, detailed data analysis for anthropometric templates, and osteotomy simulation can be viewed directly. The first step in screw placement for cervical pedicle fixation is to find an accurate entrance point. There are many measurements of cervical vertebrae and pedicles through different methods [23], [25], [27], [30], [32]. Variability in the dimensions of cervical vertebrae are summarized in Tables S1 and S2 (electronic supplementary material), which compare our results with previous studies [13], [17], [24]–[26], [30], [31], [35].

Vertebrae dimensions determine the operating space of ATPS, screw position in the transverse, vertical section and the width of anterior plate. In our study, the mVBD ranged from 11.24 to 21.93 mm in C3 to C7, similar to Koller [12] (13.83–21.60 mm), Xu et al [26] (15.42–17.89 mm), and Liu et al [30] (16.88–19.53 mm). The ranges of mVBW was 15.67 to 32.78 mm and had an increasing trend in C3 to C7 (Table 2), lower than Koller (18.13–50.62 mm). Compared with SH [17], the results of each level were greater in our measurements. aVBH provides screw placement space in the vertical plane. Our results were (8.27–17.01 mm) relative smaller, compared to Koller (11.70–32.45 mm) and Xu (14.65–16.00 mm). The results in our study showed that, although the cervical vertebrae are different from those in populations studied by Koller and Xu, there is still enough working space. These differences may have been caused by racial differences or by variations or number of specimens. Statistically significant differences in aVBH, mVBW and mVBD, which were also observed immerged data from both genders, similar to previous studies [17], [30].

Pedicle anatomy for various races, gender and different levels has shown to vary significantly. Liu et al [13] reported on the measurements of 1311 partial and complete cervical spines using meta-analysis for different races comparisons. The results found that significant differences between males and females existed at the outer pedicle width and height of C3 to C7 in the European/American population. There are more significant differences comparing the cervical pedicles of males and females in the European/American population than that exists in the Asian population (specifically in pedicle width and height). Our results show that differences of OPW and OPH existed at C3 to C6 between males and females which are similar to findings from Liu et al study. The mean OPW and OPH in our results varied in the middle-low cervical vertebrae with significant increases from cephalad C3 to caudad C7, ranging from 5.81–7.49 mm in width, and 7.77–8.69 mm in height, respectively (Tables 3), similar to the previous reports [31], [36]. In the current study, there were no significant differences between left and right OPW and OPH, as has been reported in literature [37], [38] and the OPW was found to be larger in males than in females [37], [39]. Koller et al [12] noted that pedicle height was greater than its width for both left and right pedicles of each vertebra, resembling similar observations compared to our study. In the transpedicular screw fixation technique, the dimensions of the screw are critical. The rate of pedicle wall perforation and nerve root damage will increase when the pedicle diameter is less than 4.5 mm [27]. Our results showed that the minimum diameter of OPW was 4.41 mm in males and 4.22 mm in females (Table 3). The results demonstrated that the pedicle might differ individually, so the dimensions of the screw should be appropriate for individual selection. Biomechanical tests show that the diameter of OPW suitable for rigid fixation is at least 3.5 mm. The minimum value of OPW is larger than 4 mm in C3 to C5, and 5 mm in C6 to C7 signifying an easy placement of screws with defined trajectory. The decreasing trend of OPW lower than 5 mm in C3 to C7 was lower than those reported by Chazono [39] and Kareijovic [40]. Taking into account the means and calculating the frequencies depicts that ATPS fixation using 3.5–4.0 mm diameter screws would be appropriate at all levels only in selected patients, but feasible in most of the biomechanically challenged end-levels (C6–C7) of multilevel cervical constructs.

For the mean PAL, Our mean values were 33.40–31.13 mm, similar to previous studies [12], [13], [26], [31], [36]. Compared to Wang [31], our results were probably more relevant and accurate due to our larger sample sizes. The biomechanical test also verified that the screw head and length engaging at least two third of the pedicle had more advantages. So the pedicle axial length of 21–24 mm for C3 to C7 is recommended for both males and females.

We believe that safe transpedicular screw placement in the cervical spine depends on the selection of the entry point for screw insertion and on proper orientation of the screw in the transverse and sagittal plane. The risk of violating the transverse foramen or spinal canal and intervertebral disc will depend on the TPA and SPA. Measuring the TPA using conventional imaging technique remains a challenge [30]. In one of the previous study, the TPA measured for pCPS insertion varies between a minimum mean of 36 degrees for C7 pedicle and to a maximum mean of 49 degrees for C4 pedicle [40], larger than our results which showed TPA to be 32.26 for C7 to 46.79 degrees for C3. However, the mean value is 41 degrees in our results similar to Wang [31] (43.25 degrees) but different from Koller [12] (48 degrees) and Xu [26] (47 degrees). There was a decreasing tendency in C3 to C7 for TPA, values in C3 to C5 were relatively consistent, but many differences were observed in C6 and C7 due to pedicle cohesion. The results of TPA in our results are similar to previous studies in C3 to C5 [12]. However, they vary for C6 and C7 and this could be due to racial differentiation and sample sizes. Based on these results, we recommend placement of screws with TPA of 46.79–49.00 degrees in C3 to C5, and 40.89–32.26 degrees in C6 to C7 are recommended for Chinese population.

We measured the l/rSPA formed by the pedicle axis and a line drawn along the anterior vertebral body, as this angle would be that created between an ATPS and the anterior cervical plate, our results are correspond to those reported in the literature [12], [26]. Kareikovic et al. [40] found that C3 pedicles were directed superiorly compared with the inferior endplate, that C4 and C5 pedicles were parallel to it, and that C6 and C7 pedicles were inferiorly directed. In our study, lSPA and rSPA were lowest at the C3 level with a mean of 93.54 degrees, that is an ATPS would to be directed slightly in cephalad direction in relation to the anterior vertebral cortex at C3. As mentioned, the OPH is mainly larger than the OPW. Therefore, a steeper cephalad directed trajectory for insertion of an ATPS is possible also at this level. In addition, the sagittal intersection points resembling the entry points of ATPS at these and other levels might be chosen more caudal in reference to the superior endplate of the instrumented vertebra if necessary. Therefore, the suggestion of SPA with 93.54–106.69 degrees in C3 to C6, and 109.36–104.99 in C6 to C7 are recommended from our results.

Dl/rSIP had an increasing trend away from upper endplate (1.87–5.83 mm) and Dl/rTIP had a trend of contralateral turning ipsilateral in C3 to C7 (−2.70∼3.18 mm, Tables 4). With the measurements of the distances of the sagittal and transverse intersections (l/rSIP and l/rTIP), we assessed the theoretical entry points for ATPS in to the vertebral bodies and pedicles, respectively. Due to midline crossing of the pedicle axis, insertion of ATPS was unilaterally possible. Because lTIP and rTIP resemble the varying entry points for ATPS in the transverse plane, a static or translational plate design will have to respect the individual variations of entry points in the transverse and sagittal planes, by adjusting the hole geometry and the distances between perforations at the center of the plate.

### Future application of ATPS

Another concomitant development that complements this study is the use of computer assisted orthopedic surgery (CAOS) in these surgeries. Accurate screw position was significantly improved with the advent of CAOS. It helped to standardize optimal screw placement and enabled minimally invasive approaches for surgeries (MIS). In recent years, a series of technological improvements have increased the accuracy rate of cervical screw placement, mainly through the use of preoperative multislice spiral CT [12], [23], [24] and intraoperative navigation systems [41]–[43]. Koller et al [20] analyzed the impact of using a navigation system on the accuracy of ATPS insertion and revealed an astonishingly high accuracy for the ATPS group with no critical screw position (0%) in axial or sagittal plane. This was far superior to the conventional unaided surgical technique that had a rate of accuracy of 78.3% (coronal section) and 95.7% (vertical plane). Kotani et al [44] completed a retrospective analysis of 180 pedicle screws, and significant differences were found between 6.7% using CT and 1.2% using navigation system. Ito et al [45] found that in surgery on 171 cervical pedicles using a navigation system, the rate of perforation was only 2.8%. In addition, technology based on Mimics could be used for rapid prototyping (RP) which might be used synchronously to develop personalized navigation templates. Fu et al [46] constructed a patient-specific biocompatible drill template using Mimics which suite for anterior part of cervical vertebrae after 3D reconstruction. Combining with rapid prototyping (RP) printing, the authors found that the screws in non-critical position were 44/48 (91.7%) and those in a critical position were 4/48 (8.3%) after 3.5 mm-diameter screw insertion. These personalized navigation templates will avoid the disadvantages of CAOS, particularly increased surgical time, steep learning curve and cost.

Although ATPS is a three-column fixation device that could be a valuable tool in a surgeon's armamentarium compared to other methods, the authors think that only surgeons experienced in transpedicular screw fixation and surgery of the cervical spine should perform this method of instrumentation. Thus, conventional methods of inserting pedicle screws are still important for a safe and current procedure, particularly for the use of LMS which is most frequently performed for comprehensive clinical practice in posterior cervical instrumentation [2], [7], [47]–[49].

### Limitations of this study

Although this study has several impacts, there are some limitations. First, it may be pointed out that the sample size in our study was relatively small; however, the statistical analyses have brought out significant outcomes with a scientific basis. Second, due to limitations of the function of Mimics software, although the axial line of the pedicle can be calculated by fitting, artificial selection was also needed for ensuring the optimal screw placement in the narrowest part of the pedicle. More advanced techniques like rapid prototyping may be used in conjunction with Mimics to develop real 3-D models for surgical simulation. Third, all the inputs were from CT scan DICOM data that are subject to slice thickness, slice interval, and may have some effect on actual processing by Mimics. Lastly, as this study is largely anatomical and morphometric, it is not a substitute to surgical and clinical acumen [20], [26].

## Conclusions

This study provides valuable data for ATPS in the cervical spine region for the Chinese population, suggesting that this technique is also clinically possible in selected vertebrae and patients. Based on the results of this study, combined with a wide cancellous working area inside the vertebral body [20], Morphological considerations in favor of ATPS at C3 to C7 are as follows: The entrance points for pedicular screw insertion for C3 to C5 were recommended −2∼−3 mm from the median sagittal plane, 1–4 mm from the upper endplate, with TPA being 46.79–49.00 degrees and SPA being 93.54–106.69 degrees. The entry points in the ipsilateral pedicle for the screws for C6 and C7 were 0–4 mm from the median sagittal plane, 5–6 mm from the upper endplate, with TPA being 40.89–32.26 degrees and SPA being 109.36–104.99 degrees. The pedicle screw insertion diameter was recommended 3.5 mm (C3 and C4), 4.0 mm (C5 to C7), and the pedicle axial length was 21–24 mm for C3 to C7 for both males and females. Nevertheless, it appears to us that morphological guidelines are not sufficient to provide safe space for C3 and should be individualized given its relatively smaller anatomy structure.

## Supporting Information

Table S1 **Summary of previous studies (Part 1).**
(DOC)

Table S2 **Summary of previous studies (Part2).**
(DOC)
