# Supplementary material for: CT Morphometric Analysis to Determine the Anatomical Basis for the Use of Transpedicular Screws during Reconstruction and Fixations of Anterior Cervical Vertebrae
Source: PLoS One. 2013 Dec 11;8(12):e81159. doi: 10.1371/journal.pone.0081159 (PMC3859485; doi:10.1371/journal.pone.0081159)
Supplement: Table S1 — Summary of previous studies (Part 1). (DOC) [file pone.0081159.s001.doc]

**Table S1.** Summary of previous studies (Part 1).

| level  C3 |  | aVBH (mm) | mVBW (mm) | mVBD (mm) |
| --- | --- | --- | --- | --- |
|  | Xu 2011[26] | 15.97±2.02 | 20.92±4.10 | 15.42±3.15 |
|  | Liu 2001 [30] | 14.00±1.40 | 20.29±1.41 | 16.88±1.23 |
|  | Tan 2004 [17] | 14.37 | 15.18 | 15.9 |
| C4 | Xu | 15.13±1.63 | 22.35±4.03 | 16.09±3.44 |
|  | Liu | 13.41±1.52 | 22.71±1.64 | 17.17±1.58 |
|  | Tan | 14.46 | 16.25 | 16.14 |
| C5 | Xu | 14.65±1.70 | 23.41±5.12 | 16.26±2.44 |
|  | Liu | 12.92±1.42 | 24.76±1.46 | 17.81±1.32 |
|  | Tan | 13.27 | 18.19 | 16.84 |
| C6 | Xu | 14.65±1.70 | 24.49±4.92 | 16.43±2.77 |
|  | Liu | 13.06±1.54 | 25.03±2.17 | 19.25±1.44 |
|  | Tan | 13.55 | 19.29 | 17.07 |
| C7 | Xu | 16.00±2.22 | 28.67±4.92 | 17.89±2.03 |
|  | Liu | 14.73±1.35 | 27.03±2.05 | 19.53±1.61 |
|  | Tan | 14.97 | 20.28 | 17.63 |
