# Supplementary material for: CT Morphometric Analysis to Determine the Anatomical Basis for the Use of Transpedicular Screws during Reconstruction and Fixations of Anterior Cervical Vertebrae
Source: PLoS One. 2013 Dec 11;8(12):e81159. doi: 10.1371/journal.pone.0081159 (PMC3859485; doi:10.1371/journal.pone.0081159)
Supplement: Table S2 — Summary of previous studies (Part2). (DOC) [file pone.0081159.s002.doc]

**Table S2. Summary of previous studies (Part2).**

|  |  |  | OPW (mm) | DSIP(mm) | | OPH(mm) |  | | DTIP(mm) | TPA(degrees) | |  | SPA(degrees) | PAL(mm) |
| --- | --- | --- | --- | --- | --- | --- | --- | --- | --- | --- | --- | --- | --- | --- |
| C3 | | Xu 2011 [26] | 4.82±0.60 | 3.4±0.6 | 6.1±0.64 | |  | 3.98±1.6 | | | 45.7±4.0 |  | 93.4±7.2 | 31.5±1.42 |
|  | | Wang 2012 [31] | 4.97±1.07 | 6.33±0.82 | | 7.32±0.85 | | | 2.61±0.73 | 46.68±2.43 | | | -11.44±2.89 | 29.64±1.73 |
|  | | Liu2010[13](Asian/Euro | 5.26±0.57/5.17±0.31 | NA | | 6.70±0.85/5.788±4.55 | | | NA | 48.41±1.83/47.06±1.66 | | | NA | 29.17±0.83/33.34±0.96 |
|  | | Kayalioglu 2007 [25] | 4.16±0.76 | NA | | 5.93±0.87 | | | NA | NA | | | NA | 30.54±2.48 |
|  | | Zhu 2008 [24] (M/F) | 5.4±0.6/4.4±1.0 | NA | | 6.5±0.5/5.7±0.4 | | | NA | 50.2±2.4/49.1±2.1 | | | NA | 29.6±1.0/28.2±1.1 |
|  | | Takeshi 2004 [35] (M/F) | 6.9±0.8/5.5±0.3 | NA | | NA | | | NA | 51.1±3.56/48.6±4.5 | | | NA | 30.6±1.3/29.5±1.5 |
| C4 | | Xu | 4.85±0.88 | 3.6±0.4 | | 6.5±0.53 | | | 3.26±0.9 | 50..8±5.2 | | | 100.2±7.4 | 32.4±3.4 |
|  | | Wang | 5.13±1.11 | 6.57±0.67 | | 7.25±0.90 | | | 2.54±0.54 | 47.02±2.85 | | | -7.42±3.09 | 31.28±1.15 |
|  | | Liu(Asian/Euro) | 5.33±0.60/5.31±0.37 | NA | | 6.78±0.88/6.96±0.52 | | | NA | 50.58±1.72/48.78±2.10 | | | NA | 28.90±0.95/32/81±1.24 |
|  | | Kayalioglu | 4.57±0.74 | NA | | 6.24±0.79 | | | NA | NA | | | NA | 32.4±2.54 |
|  | | Zhu(M/F) | 5.3±0.6/4..4±0.9 | NA | | 6.4±0.5/5.9±0.8 | | | NA | 51.3±2.5/52.1±3.0 | | | NA | 28.9±1.1/27.9±1.2 |
|  | | Takeshi(M/F) | 6.8±0.7/5.7±0.4 | NA | | NA | | | NA | 53.2±4.6/50.7±4.4 | | | NA | 30.3±1.5/29.5±1.4 |
| C5 | | Xu | 5.46±1.00 | 5.1±0.9 | | 6.5±0.72 | | | 2.13±1.3 | 52.1±5.9 | | | 104.5±7.2 | 32.1±1.9 |
|  | | Wang | 5.76±1.36 | 2.34±1.02 | | 7.34±1.15 | | | 47.97±1.98 | 47.97±1.98 | | | 1.16±1.23 | 30.88±0.98 |
|  | | Liu(Asian/Euro) | 5.68±0.55/5.66±0.30 | NA | | 6.95±0.49/6.74±0.45 | | | NA | 48.95±1.56/48.35±2.10 | | | NA | 30.82±0.33/33.79±1.78 |
|  | | Kayalioglu | 5.03±0.72 | NA | | 6.29±1.1 | | | NA | NA | | | NA | 32.53±2.67 |
|  | | Zhu(M/F) | 5.6±0.6/5.1±0.7 | NA | | 6.8±0.6/6.4±0.8 | | | NA | 50.9±2.3/48.3±2.7 | | | NA | 31.3±1.1/30.0±1.3 |
|  | | Takeshi(M/F) | 6.8±0.5/6.1±0.7 | NA | | NA | | | NA | 52.1±4.5/49.0±3.2 | | | NA | 31.2±1.7/30.2±2.6 |
| C6 | | Xu | 5.92±1.00 | 6.6±0.8 | | 6.3±1.11 | | | -1.27±2.1 | 47.8±6.7 | | | 112.1±6.2 | 32.5±2.3 |
|  | | Wang | 6.19±0.99 | 7.81±0.98 | | 7.29±1.06 | | | 4.21±1.31 | 40.75±3.06 | | | 8.34±2.32 | 32.03±1.42 |
|  | | Liu(Asian/Euro | 5.91±0.51/5.99±0.43 | NA | | 7.25±0.64/6.71±0.43 | | | NA | 44.94±2.03/44.24±2.14 | | | NA | 31.67±0.59/34.23±1.80 |
|  | | Kayalioglu | 5.28±0.93 | NA | | 6.23±0.97 | | | NA | NA | | | NA | 32.45±2.82 |
|  | | Zhu(M/F) | 5.8±0.6/5.4±0.7 | NA | | 6.9±0.6/6.7±0.9 | | | NA | 48.6±2.7/46.9±2.8 | | | NA | 31.2±1.3/30.8±1.3 |
|  | | Takeshi(M/F) | 7.2±0.8/6.1±0.7 | NA | | NA | | | NA | 46.1±5.1/43.7±5.7 | | | NA | 33.2±2.4/30.5±2.4 |
| C7 | | Xu | 6.61±0.71 | 7.5±0.9 | | 7.0±0.68 | | | -1.97±1.2 | 44.4±8.3 | | | 102.7±8.5 | 31.7±1.4 |
|  | | Wang | 7.07±1.03 | 8.07±1.14 | | 7.33±1.07 | | | 4.75±1.12 | 35.88±2.83 | | | 12.82±3.16 | 33.51±1.53 |
|  | | Liu(Asian/Euro) | 6.63±0.49/6.64±0.67 | NA | | 7.63±0.81/6.93±0.78 | | | NA | 37.05±3.35/38.68±4.04 | | | NA | 31.87±1.66/34.23±1.80 |
|  | | Zhu(M/F) | 6.7±0.7/6.3±0.7 | NA | | 7.2±0.7/6.9±0.9 | | | NA | 41.2±3.0/40.6±3.5 | | | NA | 32.5±1.1/31.7±1.3 |
|  | | Takeshi(M/F) | 7.7±0.9/7.0±0.9 | NA | | NA | | | NA | 35.6±5.4/33.9±3.2 | | | NA | 31.7±2.4/27.7±2.4 |
